# Tumor cell enrichment by tissue suspension enables detection of mutations with low variant allele frequency and estimation of germline mutations

**DOI:** 10.1038/s41598-022-06885-2

**Published:** 2022-02-22

**Authors:** Keiichi Hatakeyama, Koji Muramatsu, Takeshi Nagashima, Yuichi Kawanishi, Ryutaro Fukumura, Keiichi Ohshima, Yuji Shimoda, Hirotsugu Kenmotsu, Tohru Mochizuki, Kenichi Urakami, Yasuto Akiyama, Takashi Sugino, Ken Yamaguchi

**Affiliations:** 1 Medical Genetics Division, Shizuoka Cancer Center Research Institute, Sunto-gun, Shizuoka, 411-8777 Japan; 2 Division of Pathology, Shizuoka Cancer Center Research Institute, Sunto-gun, Shizuoka, 411-8777 Japan; 3 Cancer Diagnostics Research Division, Shizuoka Cancer Center Research Institute, Sunto-gun, Shizuoka, 411-8777 Japan; 4 SRL Inc, Shinjuku-ku, Tokyo, 163-0409 Japan; 5 SRL & Shizuoka Cancer Center Collaborative Laboratories Inc, Sunto-gun, Shizuoka, 411-8777 Japan; 6 Division of Genetic Medicine Promotion, Shizuoka Cancer Center, Sunto-gun, Shizuoka, 411-8777 Japan; 7 Immunotheraphy Division, Shizuoka Cancer Center Research Institute, Sunto-gun, Shizuoka, 411-8777 Japan; 8 Shizuoka Cancer Center, Sunto-gun, Shizuoka, 411-8777 Japan

**Keywords:** Biotechnology, Cancer

## Abstract

Targeted sequencing offers an opportunity to select specific drugs for cancer patients based on alterations in their genome. However, accurate sequencing cannot be performed in cancers harboring diffuse tumor cells because of low tumor content. We performed tumor cell enrichment using tissue suspension of formalin-fixed, paraffin-embedded (FFPE) tissue sections with low tumor cell content. The enriched fractions were used to efficiently identify mutations by sequencing a target panel of cancer-related genes. Tumor-enriched and residual fractions were isolated from FFPE tissue sections of intestinal and diffuse gastric cancers harboring diffuse tumor cells and DNA of suitable quality was isolated for next-generation sequencing. Sequencing of a target panel of cancer-related genes using the tumor-enriched fraction increased the number of detectable mutations and variant allele frequency. Furthermore, mutation analysis of DNA isolated from tumor-enriched and residual fractions allowed us to estimate germline mutations without a blood reference. This approach of tumor cell enrichment will not only enhance the success rate of target panel sequencing, but can also improve the accuracy of detection of somatic mutations in archived specimens.

## Introduction

In the treatment of cancer, limited genetic testing, such as companion diagnostics, provides cancer patients and clinicians with important information for effective selection of drugs. Recent large-scale analyses using next-generation sequencing (NGS) have revealed the relationship between gene alterations and various cancers^1–3^. Based on these findings, sequencing of multiple target gene panels using NGS provides an opportunity for further drug selection in clinical practice.

The detection of somatic mutations using NGS is affected by the tumor content in tissue samples^4^. Generally, sequencing of a target panel is performed using formalin-fixed, paraffin-embedded (FFPE) tissue sections^5^. Such samples with low tumor content can be subjected to tumor cell enrichment by macrodissection. However, for cancers, such as diffuse-type gastric cancer or lobular breast cancer, macrodissection is often unsuitable because of the diffused type of tumor cells. In many cases, especially in diffuse-type gastric cancer, the estimated content of tumor cells is below 30%^6^. Therefore, alternative tumor cell enrichment methods besides macrodissection are required for accurate detection of mutations in the sequencing of target panel of genes for various cancer types.

The targeted sequencing has two standard pipelines for detection of somatic mutations, one using blood as a reference and the other using public databases^5,7^. Although the pipeline using the databases has the advantage that FFPE tissue sections can be analyzed without the need for a blood reference, this approach entails the risk of simultaneous detection of alterations derived from germline mutations. The accuracy of detection of somatic mutations depends on public databases owing to population stratification in single nucleotide variants (SNVs), because of which false positive mutations are increased for populations with insufficient SNV information^8^. In contrast, in the pipeline using blood from the same patient from whom tissue is obtained, germline mutations can be reliably determined by subtracting mutations detected in the blood reference, resulting in the extraction of only somatic mutations upon targeted sequencing^7,9^. However, most archived specimens stored as FFPE tissues are not paired with a blood reference that could allow detection of somatic mutations based on targeted sequencing.

In the present study, we performed tumor cell enrichment using tissue suspensions prepared from FFPE tissue sections of diffuse-type and intestinal gastric cancers. DNA suitable for NGS was then extracted from FFPE tissue sections having the thickness that is commonly used in targeted sequencing. We next investigated the effect of this tumor cell enrichment on the detection of mutations. Finally, to accurately distinguish between somatic and germline mutations without a blood reference, mutations in DNA from the tumor and residual fractions isolated by tumor cell enrichment were analyzed. Our approach for tumor cell enrichment will improve the mutation detection rate by targeted sequencing and would enable accurate identification of somatic mutations.

## Results

### Tumor cell enrichment using tissue suspension

**Figure 1:**
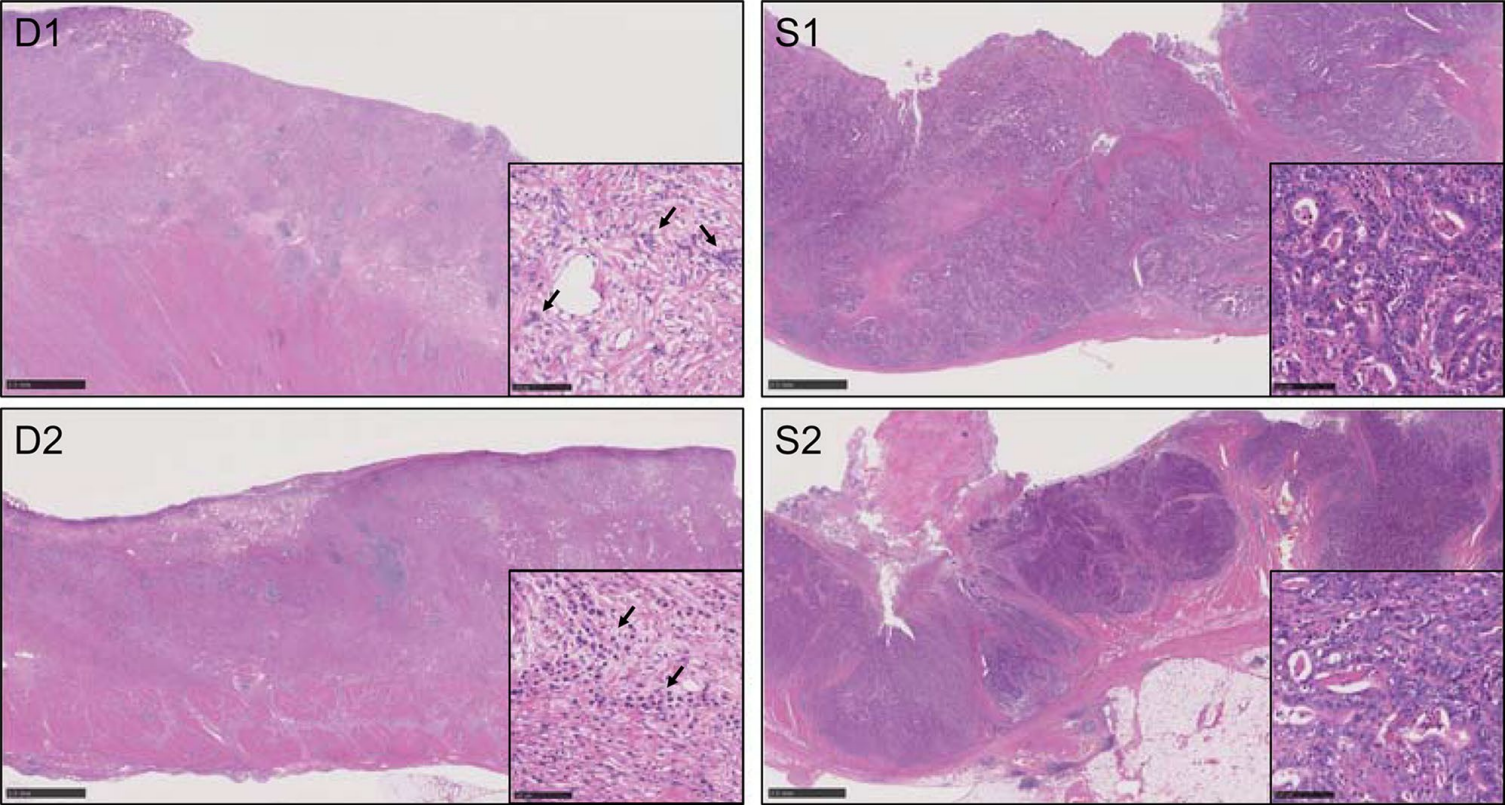
Hematoxylin and eosin (H&E) staining of gastric cancers used in next-generation sequencing. D1/2 and S1/2 were diagnosed as diffuse-type and intestinal gastric cancers, respectively. Scale bar represents 2.5 mm. Insets show a partial enlargement of the H&E-stained images. In the insets of the image for diffuse-type gastric cancer (D1 and D2), the areas with high density of tumor cells are indicated with black arrows. Scale bar in the inset represents 100 µm.

A total of 12 FFPE samples from 4 patients with gastric cancer were obtained from the tissue bank of Division of Pathology at Shizuoka Cancer Center. The series included 10, 20, and 50 µm thick FFPE tissue sections from two diffuse-type (D1 and D2) and two intestinal (S1 and S2) gastric cancers that were collected between 2014 and 2019 (Fig. 1). The tumor cellularity estimated by a pathologist was less in the diffuse-type (D1, 20%; D2, 20%) than in the intestinal type (S1, 60%; S2, 50%). These diffuse-type gastric cancers were considered unsuitable for macrodissection to enrich tumor cells in the FFPE tissue sections due to the dispersion of tumor cells in the tissue.

**Figure 2:**
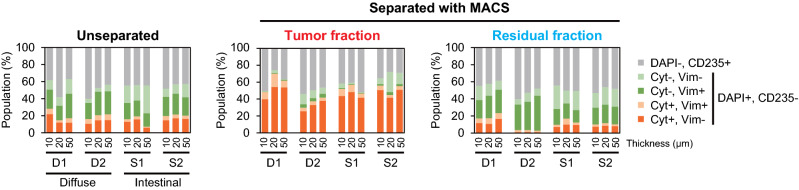
Estimation of tumor cell population in formalin-fixed paraffin-embedded (FFPE) tissue sections using flow cytometry. The FFPE tissue sections of different thickness (10, 20, and 50 µm) were suspended and separated using magnetic-activated cell sorting (MACS) with anti-cytokeratin microbeads. These fractions were stained with anti-cytokeratin and vimentin antibodies. To distinguish nuclei and erythrocytes, DAPI and CD235 staining were performed simultaneously. The suspensions enriched with the microbeads are defined as tumor fractions, and samples that could not be captured with these beads are designated as a residual fraction. The suspensions that were not subjected to MACS are designated as unseparated.

To increase the proportion of tumor cells from which DNA could be extracted in the FFPE tissue sections, tumor cell enrichment was performed using tissue suspension. The populations considered to be of tumor cells (cytokeratin + , vimentin−) were enriched in the tumor fractions compared to the unseparated samples, whereas in the residual fraction, these populations were decreased in both diffuse-type and intestinal gastric cancers (Fig. 2). The residual and tumor fractions contained 0.91–17% tumor cells and 0.37–4.4% non-tumor cells (cytokeratin-, vimentin +) as contamination, respectively.

Furthermore, no difference in the enrichment because of the thickness of the FFPE tissue sections was observed. These results indicate that tumor cells expressing cytokeratin on the cell surface could be concentrated from the FFPE tissue sections of gastric cancer with low tumor content.

### Confirmation of sample quality for sequencing

**Figure 3:**
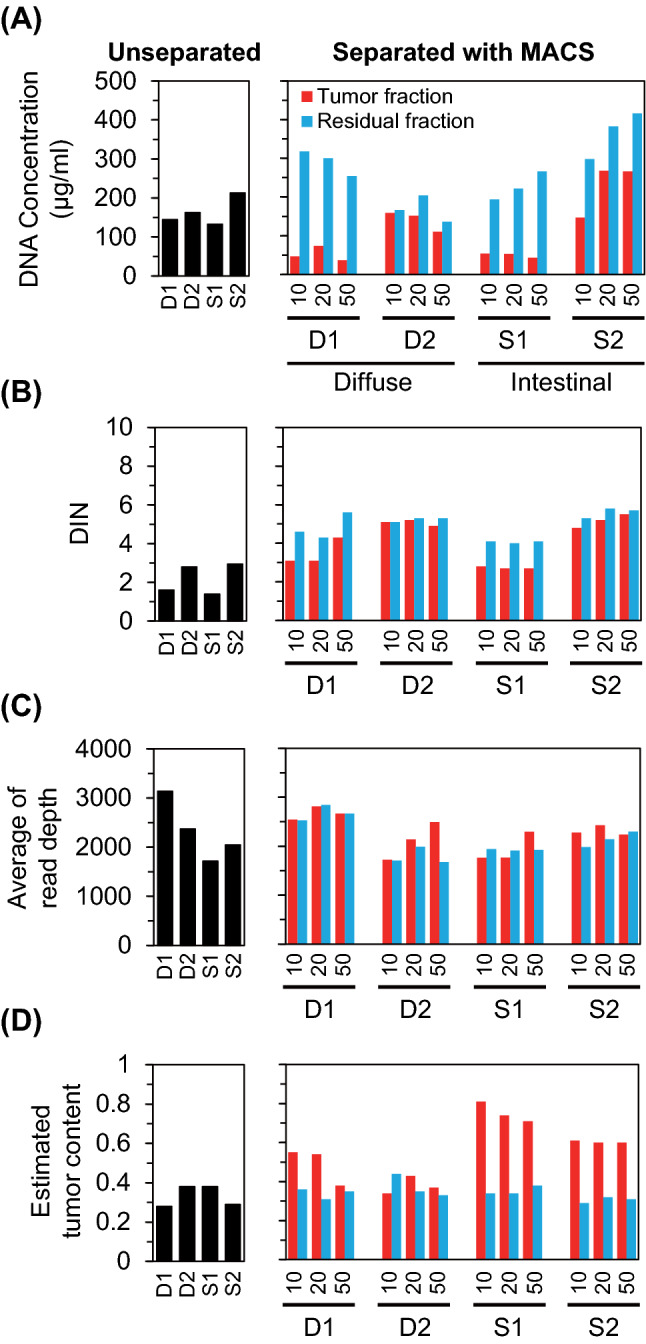
Quality of DNA, read depth, and estimated content of tumor obtained from formalin-fixed paraffin-embedded (FFPE) tissue sections. DNA concentration (**A**), DNA integrity number (DIN) (**B**), read depth (**C**), and estimated tumor content (**D**) in the FFPE tissue sections of different thickness (10, 20, and 50 µm). The suspensions enriched with the microbeads are defined as tumor fractions, and samples that could not be captured with these beads are designated as residual fractions. The samples obtained from 10 µm thick FFPE tissue sections that were not subjected to magnetic-activated cell sorting (MACS) are represented as unseparated. The numbers above the sample names in the panels for “Separated with MACS” indicate the thickness of the FFPE tissue sections.

We investigated the suitability of the quality of DNA extracted from tissue suspension samples for NGS. Based on the indicators of DNA degradation, DNA integrity number (DIN), and DNA concentration, the quality of DNA was deemed suitable for NGS (Fig. 3A,B). These samples were used for library construction and NGS. The read depth of the unseparated and separated fractions was similar (Fig. 3C). Based on NGS, the tumor content was found to be increased in most of the samples in the tumor fractions (Fig. 3D). These results suggest that NGS was properly performed from tissue-suspended samples. We conclude that NGS can be performed by tissue suspension using 10 µm thick FFPE tissue sections. Subsequent experiments were carried out with 10 µm thick sections.

### Effect of tumor cell enrichment

**Figure 4:**
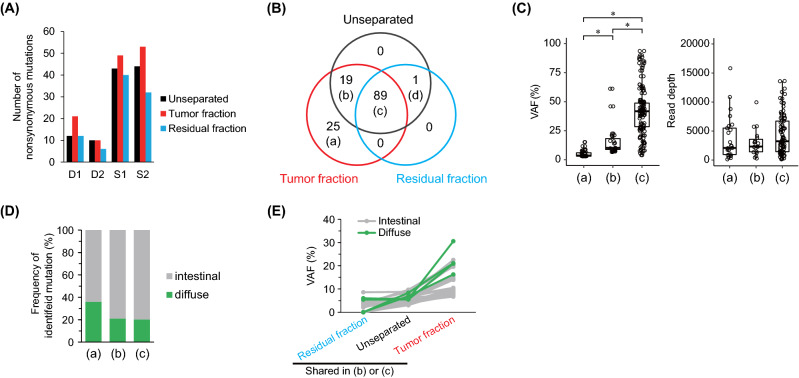
Influence of tumor cell enrichment on the detection of somatic mutations. (**A**) Comparison of nonsynonymous mutations between the separated and unseparated samples. (**B**) Venn diagram of nonsynonymous mutations detected in each separated fraction and unseparated sample. (**C**) Variant allele frequency (VAF) and read depth of somatic mutations. *p < 0.003 (Welch’s t-test with Bonferroni correction). (**D**) Frequency of somatic mutations detected in diffuse-type and intestinal gastric cancers. (**E**) Variations in the VAF in unseparated samples and tumor fractions. This variation represents mutations with less than 10% VAF in unseparated samples. Green and gray lines show variations of somatic mutations detected in diffuse-type and intestinal gastric cancers, respectively. The groups of (a), (b), and (c) correspond to the Venn diagram in (**B**). The mutation in (b) is defined as having a VAF value of 0 for the residual fraction.

To investigate whether tumor cell enrichment using the tissue suspension affects the detection of somatic mutations, we identified nonsynonymous mutations using targeted sequencing of a panel of genes (225 genes listed in Table S1 were targeted). The number of mutations detected in the tumor fraction was equal to or greater than that detected in the unseparated sample, whereas fewer mutations were detected in the residual fraction (Fig. 4A). Furthermore, 19% (25/133) of the mutations detected in the tumor fractions were tumor fraction specific (Fig. 4B). These specific mutations (a) had a significantly lower variant allele frequency (VAF) than the mutations in (b) and (c) (please see Fig. 4B for a, b, c, and d groups of mutations), although there was no difference in the read depth (Fig. 4C). These results suggest that tumor cell enrichment using the tissue suspension aided in the identification of somatic mutations that are undetected by conventional methods. Interestingly, the tumor fraction-specific mutations (a) comprised more than 30% of the mutations found in diffuse gastric cancer, implying that the tumor cell enrichment done by us contributes to better detection of mutations in this cancer type with low tumor content (Fig. 4D). For mutations that were common between the tumor fraction and unseparated samples, the low VAF was increased upon tumor cell enrichment (Fig. 4E). Because of the enrichment, 96.4% (27/28) of the identified somatic mutations with low VAF (< 10%) improved the VAF values in the tumor fraction, and the increased VAF values were, on an average, 2.4-fold higher.

### Estimation of germline mutations based on differences between the tumor and residual fractions

**Figure 5:**
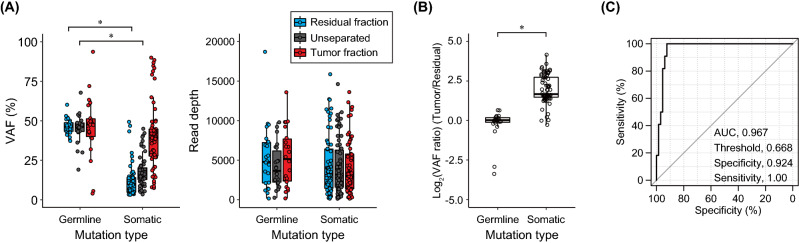
Characteristics of germline and somatic mutations in the separated and unseparated samples. (**A**) Distribution of variant allele frequency (VAF) and read depth. The genomic variations identified by whole-exome sequencing of the peripheral blood are defined as germline mutations, and the others are defined as somatic mutations. The germline mutation represents mutations found in the blood and the variant that were excluded because of their presence in the databases. (**B**) Ratio of VAF (tumor/residual) in mutations shared in all the fractions including the unseparated sample. For logarithmic transformation, the ratio is expressed as 0 when there is no change (VAF ratio = 1.0). Asterisk represents the significance (*p* < 0.01). (**C**) Receiver operating characteristic (ROC) curve for estimation of germline and somatic mutations.

The mutations detected in the sequencing of the target panel of genes excluded germline mutations present in multiple databases. Therefore, SNVs that are not registered in the databases, including those related to population differences, are identified as somatic mutations. To accurately discriminate such mutations between germline and somatic mutations, we performed whole-exome sequencing (WES) of the peripheral blood from the patient who donated the tumor tissue. In the target panel sequencing, 24 (18%) mutations were found as germline mutations (Table S2). All mutations were non-synonymous substitutions, including changes in translation start/end site and changes in splice site. The mutation designated as a germline mutation but not detected in the tumor fraction was only for one alteration in *AXL* c.1503dupC (VAF, 2.42%) in the intestinal tumor (sample S1). This mutation was excluded based on the mutation detection criterion (VAF < 3%). The VAF of evident somatic mutations based on WES of the peripheral blood was significantly decreased in the unseparated sample and residual fraction, although there was no difference in the read depth (Fig. 5A). Additionally, the evident germline mutations contained one mutation shared in the unseparated sample and residual fraction ((d) in Fig. 4B). This result raises the possibility that VAF of the evident germline mutation is independent of the tumor content in FFPE tissue sections. Based on this hypothesis, the VAF ratio of the shared mutations ((c) in Fig. 4B) was compared between the evident germline and somatic mutations. This ratio was significantly increased with true somatic mutations (Fig. 5B). Furthermore, a receiver operating characteristic (ROC) curve was generated to distinguish between somatic and germline mutations using the VAF ratios. The area under the curve (AUC) was 0.967 with the VAF ratio of 0.668 as the threshold (Fig. 5C). These results indicate that the VAF ratio using the tumor and residual fractions derived from FFPE tissue sections enables the estimation of germline mutations.

## Discussion

In this study, the intestinal and diffuse-type gastric cancer samples with low tumor content were selected by pathologists based on pathological images. To evaluate the proportion of tumor cells, these FFPE-tissue sections were suspended and stained with anti-keratin and anti-vimentin antibodies. Tanami and colleagues have reported that the intracellular expression of keratin is biased and depends on the type of gastric cancer^10^. Single-cell analysis of gastroesophageal tumors has revealed that the expression of keratin gene family and EMT-related genes is heterogeneous^11,12^. Therefore, the flow cytometric analysis using the antibodies performed in this study is unsuitable to accurately evaluate heterogeneous populations as tumor cells, leading to a discrepancy in the tumor content determined using the population analysis and that estimated by a pathologist. This discordance might be resolved by identification of proteins that are less susceptible to tumor heterogeneity and selection of antibodies against them.

The thickness of the FFPE tissue section used in clinical targeted sequencing, such as Foundation One or OncoGuide for Japanese cancer genome mutations, is 10 µm^5,7^. However, tissue cell suspensions have been prepared from FFPE tissue sections with thickness of 50 µm to maintain the cell shape^13^. We confirmed the quality of DNA extracted from FFPE tissue sections of different thicknesses, and show, for the first time, that the quality of DNA suitable for NGS is guaranteed even at a thickness of 10 µm. Furthermore, no decrease in read depth was observed in the target panel sequencing. These results suggest that sufficient DNA for NGS is retained in the slices after dewaxing and heat-induced antigen retrieval. The method of tumor cell enrichment using tissue suspension described by us is applicable to FFPE tissue sections for targeted sequencing performed in clinical practice.

Clinical FFPE tissue sections often have low tumor content, which is insufficient for identifying somatic mutations using targeted sequencing of a panel of genes. Macrodissection is commonly performed to enrich tumor cells when the tumor content of FFPE tissue sections does not meet the criteria for sequencing. Although this step is unsuitable for diffuse-type gastric cancer, which is characterized by tumor cells spread over the tissue, enrichment of tumor cells by tissue suspension, including a fully automated process, increased the number of mutations detected in these cancers. In addition, this method can increase the VAF of somatic mutations in gastric cancers; whereas no increase in tumor cells was observed in diffuse gastric cancer (sample D2). In target panel sequencing performed in clinical practice, cases with no enrichment upon macrodissection are often observed. We speculate that this is due to the heterogeneity of the tumor, but this frequency needs to be verified in the future. Hence, the tumor cell enrichment approach described by us may contribute in the improvement of the success rate of targeted sequencing of a panel of genes for cancers in which tumor cells are spread out.

Clinically, in targeted sequencing without blood sampling, germline mutations are estimated based on databases. In our study three public databases (1000 genomes project, ExAC, and gnomAD) were used. Moreover, using the peripheral blood of the same patient as a reference, 18% of the mutations found in the sequencing performed by us were distinguished as germline mutations. Based on the analysis of genetic variations using NGS, approximately 20% of the SNVs are considered to be private to the Japanese population or to a continental area^8^. Therefore, the germline mutations detected using blood as a reference are likely to reflect private SNVs that could not be excluded by the databases. Additionally, the germline mutations found using blood as a reference included a mutation ((d) in Fig. 4B) that was undetectable in the tumor fraction. The tumor fraction contains fewer normal cells than the residue fraction because of the enrichment of tumor cells by tissue suspension. Thus, it is reasonable to postulate that this mutation harboring loss of heterozygosity could only be detected in the unseparated sample and in the residual fraction. We believe that no somatic mutation was missed by tumor enrichment in this study.

Both tumor and residual fractions isolated by tissue cell suspension could be used for the NGS analysis. The estimation of mutation type using VAF of the two fractions with biased tumor content allowed us to discriminate somatic and germline mutations in samples with only FFPE tissue sections. In fact, NGS with tissue suspension of FFPE tissue sections enables the elimination of private mutations associated with racial or continental area without a blood reference. This method will contribute to the accurate estimation of personalized germline mutations in archived specimens without the use of blood samples. Although we demonstrate the enrichment concept in a small number of cases, a larger sample size is needed in the future to validate the reproducibility. Unfortunately, there are few diffuse gastric cancers with a high frequency of somatic mutations for validation in the cohort. To ensure reproducibility for adaptation to various carcinomas with low tumor content, analysis is necessary to include other cancers in which tumor cells are spread out in addition to diffuse gastric cancer in future.

In the present study, we developed a tumor cell enrichment method using tissue suspension to efficiently identify mutations in target panel sequencing from FFPE tissue sections with low tumor content. Tumor and residual fractions were isolated from intestinal gastric cancer and diffuse-type gastric cancer with diffuse tumor cells using magnetic separation after FFPE tissue suspension, and DNA having NGS-ready quality was extracted from a 10 µm thick section that is commonly used in targeted sequencing. The enrichment of tumor cells from gastric cancers increased the number of mutations identified and contributed to the improvement in the VAF. Furthermore, mutation analysis using tumor and residual fractions allowed us to estimate germline mutations without a blood reference. Our approach will not only contribute to enhancing the success rate of target panel sequencing through tumor enrichment, but also has promising prospects for improving the accuracy of detection of somatic mutations in archived specimens.

## Methods

### Ethical statement

Written informed consent was obtained from all patients, and all aspects of this study were approved by the Institutional Review Board of Shizuoka Cancer Center (authorization number 25–33). In this study, pathogenic germline mutations could be unintentionally predicted from retrospective FFPE specimens. To avoid disadvantaging specimen donors, we implemented appropriate informed consent with the approval of the Ethics Review board, including the possibility of secondary findings, such as those found in blood-based constitutional analysis. All the experiments using clinical samples were performed in accordance with the approved Japanese ethical guidelines (Human Genome/Gene Analysis Research, 2017, provided by the Ministry of Health, Labor, and Welfare; https://www.mhlw.go.jp/stf/seisakunitsuite/bunya/hokabunya/kenkyujigyou/i-kenkyu/index.html).

### Clinical samples

Two diffuse-type and two intestinal gastric cancers were extracted from the Japanese pan-cancer cohort (project HOPE) comprising 5521 tumor specimens^3^. These samples were clinicopathologically diagnosed by a pathologist after surgery. Tumors were dissected from surgical specimens immediately after resection of the lesion at the Shizuoka Cancer Center Hospital, and then the specimens were stored as FFPE tissue. In addition, peripheral blood was collected as a paired control to exclude germline mutations. Details of experimental protocols have been previously described^3,6,9,14–16^. Briefly, DNA was extracted from tissues and peripheral blood samples using a QIAamp DNA Blood Mini Kit (Qiagen, Venlo, The Netherlands). Purified DNA was quantified using a NanoDrop and Qubit 2.0 Fluorometer (Thermo Fisher Scientific, Waltham, MA).

### Dissociation and suspension of FFPE tissue samples

In the cohort, we selected relatively new samples with low tumor content and high tumor mutational burden (TMB) for whole exome sequencing to avoid the effect of DNA degradation. A FFPE tissue block of gastric cancers was cut into 10, 20, and 50 μm thick sections. These sections were dewaxed by 10 min incubation in xylene thrice and then rehydrated by 30 s incubation sequentially in each of the following dilutions of ethanol: 100% (two times), 70%, 50%, and 30%. The hydration process was completed with 30 s incubations in deionized water. The dewaxed samples were suspended using gentleMACS Octo Dissociator with Heaters (program, 37C_FFPE_1; Miltenyi Biotec, Bergisch Gladbach, Germany), after heat-induced antigen retrieval was performed according to the manufacturer’s protocol.

### Isolation and staining of cells

Fully automated cell labeling and separation was performed using the autoMACS Pro Separator (Miltenyi Biotec) according to the manufacturer’s protocol. The cell suspensions derived from FFPE tissue sections were separated using the Anti-Cytokeratin MicroBeads (Miltenyi Biotec), and were stained using the anti-cytokeratin-FITC (clone REA831, Miltenyi Biotec), anti-vimentin-APC (clone REA409, Miltenyi Biotec), and CD235a (Glycophorin A)-PE (clone REA175, Miltenyi Biotec) antibodies. The nuclei were stained with the DAPI Staining Solution (Miltenyi Biotec).

### DNA isolation

DNA was extracted from FFPE tissue and peripheral blood samples using a GeneRead DNA FFPE Kit and QIAamp DNA blood Mini Kit (Qiagen), respectively. Purified DNA was quantified using a NanoDrop and Qubit 2.0 Fluorometer (Thermo Fisher Scientific). To check the quality of DNA, DIN was determined using TapeStation (Agilent Technologies, Santa Clara, CA).

### Targeted sequencing of the gene panel

For targeted sequencing genes in DNA isolated from the FFPE tissue, a library of 225 genes (listed in Table S1) was constructed using a hybridization-based enrichment protocol (SureSelect Custom panel, Agilent). In total, 2.427 Mb of the human genome, including 0.723 Mb exon regions of RefSeq genes, were encompassed by 55,765 biotinylated RNA oligomers (120 bp length). Binary raw data derived from the sequencer were converted into sequence reads using bcl2fastq (ver. 2.20, Illumina) that were mapped to the reference human genome (UCSC hg19). Genomic alterations were identified using VarDictJava (https://github.com/AstraZeneca-NGS/VarDictJava)^17^. To reduce false-positive findings, mutations fulfilling any of following criteria were eliminated: (1) quality score < 20; (2) depth of coverage < 100; (3) depth of coverage for the alternate allele < 5; (4) VAF < 0.5%; (5) not fitting the filtering criteria of the variant caller (the FILTER field of the VCF record was not “PASS”). After annotating the mutations, those with an allele frequency of 1% or more in any of the databases shown below were excluded as common SNVs: (1) the 1000 genomes project (global or East Asia); (2) ExAC; (3) gnomAD. In addition, mutations that appeared to affect protein structure, namely missense variants, splice acceptor variants, splice donor variants, splice region variants, stop-gain variants, stop-lost variants, stop-retained variants, 5′-untranslated region premature start codon gain variants, exon-loss variants, disruptive inframe deletions, disruptive inframe insertions, frameshift variants, inframe deletions, inframe insertions, or initiator codon variants, were extracted. To ensure reproducibility of the sequencing, mutations with VAF ≥ 3% were defined as valid mutations. The tumor content was estimated by All-FIT algorithm based on tumor-only sequencing data^18^. All mutations identified as somatic were manually verified using the Integrative Genomics Viewer (IGV, https://software.broadinstitute.org/software/igv/).

### Whole-exome sequencing

To accurately distinguish germline mutations without an estimation based on databases, we used a pipeline constructed by us^3^. In brief, the exome library was constructed using an Ion Torrent AmpliSeq RDY Exome Kit (Thermo Fisher Scientific). The exome library supplied 292,903 amplicons covering 57.7 Mb of the human genome, comprising 34.8 Mb of exonic sequences from 18,835 genes registered in RefSeq. Raw binary data produced by sequencers were processed using the Torrent Suite Software (ver.5, Thermo Fisher Scientific). Processed sequence reads were mapped to the reference human genome (UCSC hg19) and genomic alterations were identified using the Torrent Variant Caller (ver.5, Thermo Fisher Scientific). To avoid sequencer- and amplicon-derived errors, arbitrary somatic mutations (VAF ≥ 10%) were manually inspected using the IGV, and somatic mutation candidates containing multiple nucleotide variations (~ 1000 sites) were validated by Sanger sequencing.

### Statistical analysis

A significant difference in read depth and VAF (including VAF ratio) was determined using the Welch's t-test. Bonferroni correction was performed for multiple comparisons. A *P*-value < 0.01 was considered significant.

## Supplementary Information

Supplementary Information.

## Data Availability

The authors declare that all the other data supporting the findings of this study are available within the article and its supplementary information files and from the corresponding author upon reasonable request.
