# Supplementary material for: Tumor cell enrichment by tissue suspension enables detection of mutations with low variant allele frequency and estimation of germline mutations
Source: Sci Rep. 2022 Feb 22;12:2953. doi: 10.1038/s41598-022-06885-2 (PMC8863826; doi:10.1038/s41598-022-06885-2)
Supplement: Supplementary file 1 — Supplementary Information. [file 41598_2022_6885_MOESM1_ESM.docx]

**Tumor cell enrichment by tissue suspension enables detection of mutations with low variant allele frequency and estimation of germline mutations**

Keiichi Hatakeyama^1,*^, Koji Muramatsu^2^, Takeshi Nagashima^3,4^, Yuichi Kawanishi^5^, Ryutaro Fukumura^5^, Keiichi Ohshima^1^, Yuji Shimoda^3,4^, Hirotsugu Kenmotsu^6^, Tohru Mochizuki^1^, Kenichi Urakami^3^, Yasuto Akiyama^7^, Takashi Sugino^2^ and Ken Yamaguchi^8^

^1^Medical Genetics Division, Shizuoka Cancer Center Research Institute, Sunto-gun, Shizuoka 411-8777, Japan

^2^Division of Pathology, Shizuoka Cancer Center Research Institute, Sunto-gun, Shizuoka 411-8777, Japan

^3^Cancer Diagnostics Research Division, Shizuoka Cancer Center Research Institute, Sunto-gun, Shizuoka 411-8777, Japan

^4^SRL Inc., Shinjuku-ku, Tokyo 163-0409, Japan

^5^SRL & Shizuoka Cancer Center Collaborative Laboratories Inc., Sunto-gun, Shizuoka 411-8777, Japan

^6^Division of Genetic Medicine Promotion, Shizuoka Cancer Center, Sunto-gun, Shizuoka 411-8777, Japan

^7^Immunotheraphy Division, Shizuoka Cancer Center Research Institute, Sunto-gun, Shizuoka 411-8777, Japan

^8^Shizuoka Cancer Center, Sunto-gun, Shizuoka 411-8777, Japan

* Correspondence should be addressed to Keiichi Hatakeyama (email: k.hatakeyama@scchr.jp; phone: +81-55-989-5222; fax: +81-55-989-6085)


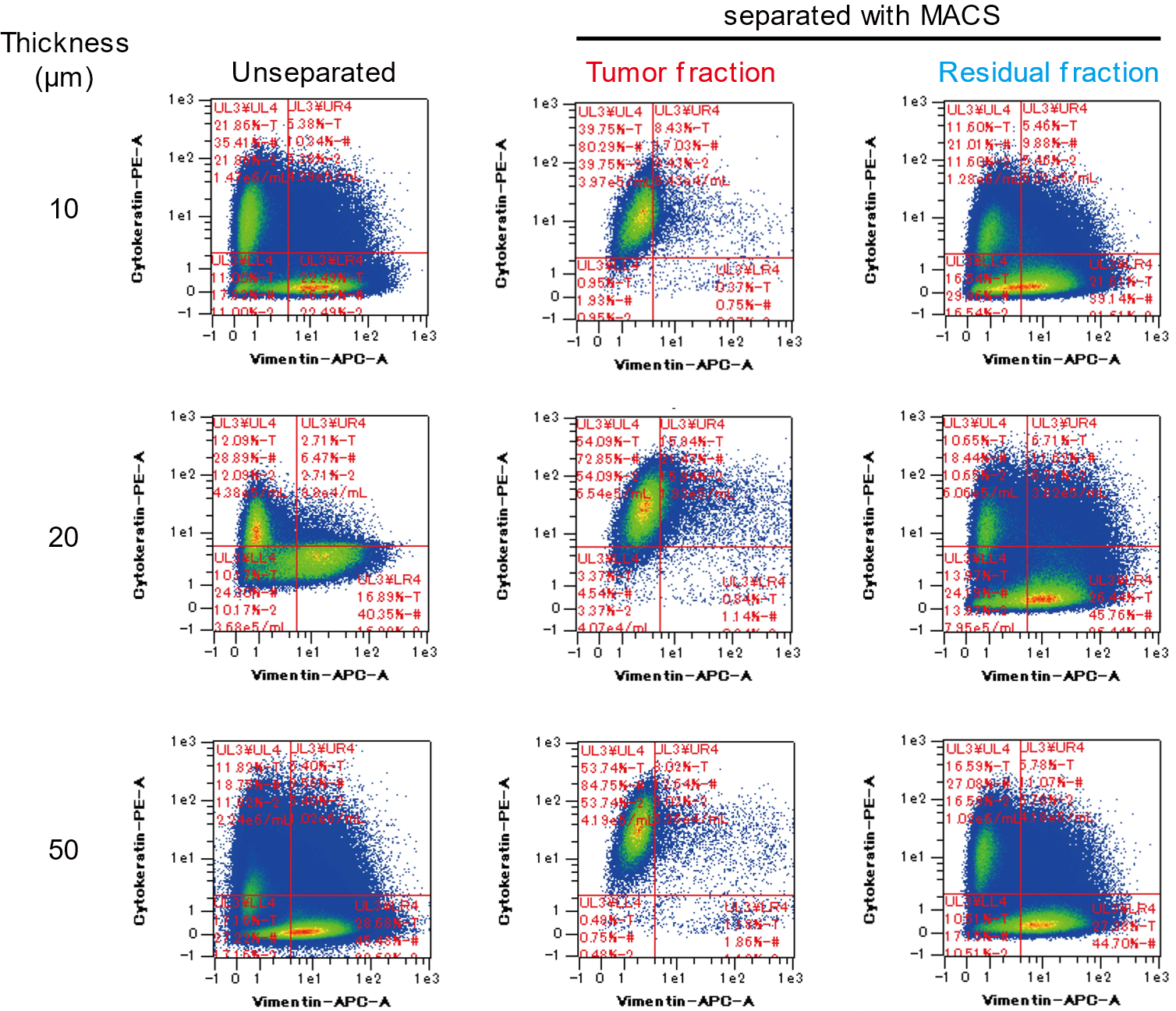


**Supplementary Figure S1.** **Flow cytometry of formalin-fixed paraffin-embedded (FFPE) tissue sections.** The results for diffuse-type gastric cancer (D1) are presented. The FFPE tissue sections of different thickness (10, 20, and 50 µm) were suspended and separated using magnetic-activated cell sorting (MACS) with anti-cytokeratin microbeads. Thereafter, these fractions were stained with anti-cytokeratin and vimentin antibodies. To distinguish nuclei and erythrocytes, DAPI and CD235 staining were simultaneously performed. The suspensions enriched with the microbeads are defined as tumor fractions, and samples that could not be captured with these beads are designated as residual fractions. The suspensions that were not subjected to MACS are represented as unseparated.

**Supplementary Table S1. List of 225 genes in target panel sequencing**

| Target gene (225 genes) | | | | | | | |
| --- | --- | --- | --- | --- | --- | --- | --- |
| *ABL1* | *CCND1* | *ENG* | *IDH1* | *MITF* | *PDGFRA* | *SDHAF2* | *TSC1* |
| *ACTN4* | *CD274* | *ENO1* | *IGF1R* | *MKRN1* | *PDGFRB* | *SDHB* | *TSC2* |
| *ACVR1B* | *CD74* | *EP300* | *IGF2* | *MLH1* | *PHOX2B* | *SDHC* | *TSHR* |
| *AKT1* | *CDC73* | *EPAS1* | *IL7R* | *MSH2* | *PIK3CA* | *SDHD* | *U2AF1* |
| *AKT2* | *CDH1* | *ERBB2* | *IRF4* | *MSH6* | *PIK3R1* | *SETD2* | *UGT1A1* |
| *AKT3* | *CDK4* | *ERBB3* | *JAK1* | *MTOR* | *PIK3R2* | *SF3B1* | *VHL* |
| *ALK* | *CDK6* | *ERBB4* | *JAK2* | *MUTYH* | *PMS2* | *SH2D1A* | *VTI1A* |
| *AMER1* | *CDKN1A* | *ERG* | *JAK3* | *MYB* | *POLD1* | *SKP2* | *WT1* |
| *APC* | *CDKN1B* | *ESR1* | *JUN* | *MYC* | *POLE* | *SMAD2* |  |
| *AR* | *CDKN2A* | *EXT1* | *KDM5C* | *MYCL* | *PPP2R1A* | *SMAD4* |  |
| *ARAF* | *CDKN2B* | *EXT2* | *KDM6A* | *MYCN* | *PRDM1* | *SMARCA4* |  |
| *ARID1A* | *CDKN2C* | *EZH2* | *KEAP1* | *MYD88* | *PRKAR1A* | *SMARCB1* |  |
| *ARID1B* | *CHEK2* | *EZR* | *KIAA1549* | *NCOA3* | *PRKCI* | *SMO* |  |
| *ARID2* | *CIC* | *FANCC* | *KIF1B* | *NCOA4* | *PTCH1* | *SOX2* |  |
| *ATM* | *COL1A1* | *FAT1* | *KIF5B* | *NCOR1* | *PTEN* | *SOX9* |  |
| *ATRX* | *CREBBP* | *FBXW7* | *KIT* | *NF1* | *PTPRK* | *SPOP* |  |
| *AXIN1* | *CRKL* | *FGFR1* | *KLF4* | *NF2* | *RAC1* | *STAG2* |  |
| *AXL* | *CRLF2* | *FGFR2* | *KMT2C* | *NFE2L2* | *RAC2* | *STAT3* |  |
| *B2M* | *CSF1R* | *FGFR3* | *KRAS* | *NFIB* | *RAD51C* | *STK11* |  |
| *BAP1* | *CTCF* | *FGFR4* | *LMO1* | *NKX2-1* | *RAF1* | *STRN* |  |
| *BARD1* | *CTLA4* | *FH* | *MAP2K1* | *NOTCH1* | *RB1* | *TACC3* |  |
| *BAX* | *CTNNB1* | *FLCN* | *MAP2K4* | *NOTCH2* | *RECQL4* | *TCF7L2* |  |
| *BCL10* | *CUL3* | *FOXL2* | *MAP3K1* | *NOTCH3* | *RET* | *TEK* |  |
| *BCL2L11* | *CYLD* | *FUBP1* | *MAP3K4* | *NRAS* | *RHOA* | *TERT* |  |
| *BMPR1A* | *DAXX* | *G6PD* | *MAPK1* | *NRG1* | *RNF43* | *TMEM127* |  |
| *BRAF* | *DDR2* | *GATA3* | *MAX* | *NTRK1* | *ROS1* | *TMPRSS2* |  |
| *BRCA1* | *DNMT1* | *GNA11* | *MDM2* | *NTRK2* | *RRAS2* | *TP53* |  |
| *BRCA2* | *DPYD* | *GNAQ* | *MDM4* | *NTRK3* | *RSPO2* | *TP63* |  |
| *CARD11* | *EGFR* | *GNAS* | *MED12* | *PALB2* | *RSPO3* | *TPM3* |  |
| *CASP8* | *EIF3E* | *HNF1A* | *MEN1* | *PBRM1* | *SALL4* | *TPMT* |  |
| *CCDC6* | *EML4* | *HRAS* | *MET* | *PDGFB* | *SDC4* | *TRAF7* |  |

**Supplementary Table S2. List of mutations identified as somatic alterations based on the databases in 10 µm thick FFPE tissue sections**

| Symbol_position Ref>Var | sample | VAF | | | depth | | | Discrimination using blood |
| --- | --- | --- | --- | --- | --- | --- | --- | --- |
|  |  | Tumor | Un-separated | Residual | Tumor | Un-separated | Residual |  |
| ACVR1B_c.1261+2T>G | D1 | 3.23 | 0 | 0 | 1983 | NA | NA | somatic |
| ACVR1B_c.652T>C | D1 | 3.02 | 0 | 0 | 5200 | NA | NA | somatic |
| ATRX_c.1492A>G | D1 | 42.44 | 47.07 | 46.3 | 1593 | 4738 | 1177 | germline |
| AXIN1_c.1597C>T | D1 | 3.09 | 0 | 0 | 15813 | NA | NA | somatic |
| BRAF_c.1406G>T | D1 | 30.6 | 5.59 | 6.03 | 1585 | 7502 | 1874 | somatic |
| BRCA1_c.2726A>T | D1 | 48.81 | 42.76 | 42.95 | 1172 | 4090 | 1411 | germline |
| CDH1_c.1321-1G>T | D1 | 79.16 | 11.65 | 9.39 | 1243 | 1872 | 2555 | somatic |
| ERBB4_c.3641A>G | D1 | 3.09 | 0 | 0 | 6109 | NA | NA | somatic |
| EZR_c.-122G>T | D1 | 3.02 | 0 | 0 | 5500 | NA | NA | somatic |
| HNF1A_c.872delC | D1 | 3.47 | 0 | 0 | 5854 | NA | NA | somatic |
| JAK2_c.3144C>A | D1 | 20.91 | 5.47 | 5.44 | 1368 | 4640 | 1158 | somatic |
| MTOR_c.61G>A | D1 | 39.27 | 12.67 | 10.05 | 5113 | 3251 | 6369 | somatic |
| PDGFRB_c.2258C>T | D1 | 51.56 | 53.1 | 50.09 | 7244 | 2030 | 6903 | germline |
| PDGFRB_c.2972G>A | D1 | 50.28 | 50.12 | 50.96 | 9234 | 2594 | 9605 | germline |
| POLD1_c.512C>T | D1 | 48.4 | 45.7 | 48.03 | 13586 | 9786 | 18716 | germline |
| PTEN_c.532_534delTAT | D1 | 6.79 | 0 | 0 | 854 | NA | NA | somatic |
| PTEN_c.968dupA | D1 | 8.13 | 0 | 0 | 123 | NA | NA | germline |
| RECQL4_c.1064G>A | D1 | 59.79 | 46.39 | 49.07 | 9828 | 1595 | 9238 | germline |
| SMARCA4_c.4210G>A | D1 | 3.55 | 0 | 2.55 | 1716 | NA | 2278 | somatic |
| TCF7L2_c.1593G>T | D1 | 57.4 | 44.95 | 49.37 | 6453 | 10068 | 6121 | somatic |
| TSC2_c.3475C>T | D1 | 43.15 | 36.26 | 45.33 | 4857 | 2780 | 7317 | germline |
| ARID1A_c.1113dupG | D2 | 46.03 | 22.86 | 0 | 252 | 280 | NA | somatic |
| KIF1B_c.4406G>A | D2 | 16.28 | 6.88 | 0 | 1241 | 1658 | NA | somatic |
| MED12_c.5429G>T | D2 | 21.14 | 8.37 | 0 | 2866 | 4016 | NA | somatic |
| NOTCH2_c.7_8delinsTT | D2 | 11.78 | 10.27 | 8.94 | 2970 | 3632 | 3108 | somatic |
| NOTCH3_c.4039G>C | D2 | 71.97 | 48.75 | 45.83 | 157 | 240 | 144 | somatic |
| PDGFB_c.35G>T | D2 | 43.96 | 40.6 | 43.31 | 2134 | 2473 | 1905 | somatic |
| PHOX2B_c.765_779delGGCAGCGGCGGCAGC | D2 | 24.51 | 19.13 | 39.59 | 971 | 1286 | 821 | somatic |
| RECQL4_c.1321C>T | D2 | 47.39 | 45.84 | 47.03 | 9908 | 9208 | 5575 | germline |
| STK11_c.437A>G | D2 | 38.59 | 39.81 | 46.79 | 3239 | 4105 | 3552 | somatic |
| TP53_c.529_546del | D2 | 61.18 | 26.03 | 2.37 | 3297 | 4936 | 4091 | somatic |
| ARID1A_c.2382dupG | S1 | 38.86 | 15.64 | 13.52 | 2831 | 2488 | 2862 | somatic |
| ARID1A_c.5548dupG | S1 | 38.23 | 17 | 12.03 | 5087 | 5870 | 6448 | somatic |
| ATM_c.1010G>A | S1 | 38.6 | 13.5 | 13.71 | 285 | 274 | 350 | somatic |
| AXIN1_c.1333C>T | S1 | 3.58 | 0 | 0 | 10850 | NA | NA | somatic |
| AXIN1_c.1523delG | S1 | 35.65 | 16.83 | 13.52 | 8489 | 10713 | 12664 | somatic |
| AXL_c.1503dupC | S1 | 2.42 | 22.41 | 25.11 | 2516 | 2566 | 3082 | germline |
| BAX_c.121delG | S1 | 81.36 | 34.33 | 24.59 | 7638 | 9338 | 10916 | somatic |
| BRAF_c.1447A>G | S1 | 29.96 | 13.22 | 11.02 | 998 | 749 | 717 | somatic |
| BRCA2_c.2957delA | S1 | 3.49 | 0 | 0 | 344 | NA | NA | somatic |
| BRCA2_c.3019G>T | S1 | 13.92 | 5.38 | 0 | 431 | 260 | NA | somatic |
| CASP8_c.1177A>G | S1 | 42.64 | 19.39 | 14.1 | 2031 | 1604 | 2007 | somatic |
| CTNNB1_c.1346G>A | S1 | 42.46 | 18.05 | 14.1 | 3375 | 3041 | 3411 | somatic |
| CYLD_c.88G>A | S1 | 4.39 | 0 | 0 | 683 | NA | NA | somatic |
| EPAS1_c.1658C>T | S1 | 3.09 | 0 | 0 | 4692 | NA | NA | somatic |
| EPAS1_c.955C>A | S1 | 5.22 | 0 | 0 | 7599 | NA | NA | somatic |
| ERBB3_c.1442G>A | S1 | 42.45 | 19.34 | 14.36 | 2641 | 2720 | 3072 | somatic |
| FAT1_c.12629A>T | S1 | 41.71 | 21.62 | 14.79 | 2201 | 2077 | 2136 | somatic |
| FAT1_c.2510T>C | S1 | 7.12 | 3.68 | 2.85 | 3116 | 2367 | 2740 | somatic |
| FAT1_c.3423G>C | S1 | 39.27 | 43.36 | 37.25 | 1416 | 1100 | 1345 | germline |
| FBXW7_c.1712G>T | S1 | 7.94 | 3.49 | 3.82 | 1411 | 1116 | 1388 | somatic |
| FGFR1_c.1052A>G | S1 | 7.49 | 6.29 | 4.66 | 2990 | 2814 | 3092 | somatic |
| FGFR3_c.2414G>A | S1 | 15.58 | 5.93 | 4.04 | 7515 | 9289 | 10098 | somatic |
| FH_c.956A>G | S1 | 10.53 | 7.13 | 4.03 | 874 | 743 | 917 | somatic |
| FLCN_c.1285delC | S1 | 38 | 19.26 | 13.13 | 7137 | 8074 | 9138 | somatic |
| GATA3_c.708delC | S1 | 14.29 | 5.01 | 3.29 | 5801 | 6985 | 7485 | somatic |
| JAK1_c.425dupA | S1 | 40.37 | 16.94 | 14.14 | 2695 | 2656 | 3105 | somatic |
| KIAA1549_c.3974G>A | S1 | 9.3 | 3.77 | 2.56 | 3872 | 3100 | 3470 | somatic |
| KIAA1549_c.5191G>C | S1 | 61.37 | 53.9 | 53.11 | 9811 | 8606 | 9487 | germline |
| MAP2K1_c.371C>T | S1 | 64.83 | 26.83 | 17.06 | 2249 | 2169 | 2679 | somatic |
| MSH6_c.407A>T | S1 | 41.82 | 18.88 | 13.21 | 1363 | 1372 | 1476 | somatic |
| NOTCH1_c.5950C>T | S1 | 37.38 | 17.72 | 13.68 | 11739 | 14612 | 15867 | somatic |
| NOTCH2_c.7_8delinsTT | S1 | 8.53 | 8.76 | 8.6 | 8011 | 9393 | 10552 | somatic |
| NOTCH3_c.3523C>T | S1 | 93.66 | 67.81 | 60.18 | 2966 | 4001 | 4422 | germline |
| PIK3CA_c.3140A>G | S1 | 50.13 | 24.1 | 21.35 | 1137 | 697 | 726 | somatic |
| PIK3CA_c.323G>A | S1 | 26.97 | 14.55 | 11.57 | 660 | 440 | 432 | somatic |
| PTCH1_c.3606delC | S1 | 44.81 | 22.46 | 16.71 | 10588 | 9652 | 11110 | somatic |
| PTCH1_c.3907C>T | S1 | 49.66 | 46.48 | 44.61 | 6053 | 6659 | 6792 | germline |
| RNF43_c.575delC | S1 | 84.83 | 35.46 | 27.48 | 2940 | 3663 | 4159 | somatic |
| SALL4_c.200G>A | S1 | 25.83 | 12.75 | 10.39 | 4302 | 4518 | 4803 | somatic |
| SALL4_c.2983delG | S1 | 28.25 | 15.85 | 11.76 | 3759 | 3173 | 3495 | somatic |
| SALL4_c.3149T>C | S1 | 31.96 | 43.34 | 42 | 2638 | 2118 | 2150 | germline |
| SMARCA4_c.2092G>A | S1 | 88.43 | 37.25 | 29.75 | 3120 | 3313 | 3526 | somatic |
| SMARCB1_c.1091_1093delAGA | S1 | 36.5 | 17.15 | 11.87 | 5737 | 6863 | 7091 | somatic |
| SOX2_c.229G>A | S1 | 3.07 | 0 | 0 | 8784 | NA | NA | somatic |
| TACC3_c.2227G>A | S1 | 47.81 | 48.43 | 46.02 | 2675 | 3285 | 3553 | germline |
| TEK_c.1250delC | S1 | 44.69 | 21.81 | 17.75 | 1289 | 1073 | 1234 | somatic |
| TEK_c.255delA | S1 | 3.1 | 0 | 0 | 1744 | NA | NA | somatic |
| TMPRSS2_c.137C>T | S1 | 44.01 | 18.93 | 14.82 | 8248 | 8068 | 9194 | somatic |
| TP53_c.91G>A | S1 | 39.29 | 16.38 | 13.83 | 761 | 995 | 1077 | somatic |
| TSC2_c.2072G>A | S1 | 43.54 | 20.14 | 14.19 | 1525 | 1822 | 2170 | somatic |
| ACTN4_c.409G>A | S2 | 7.15 | 4.83 | 0 | 4168 | 3540 | NA | somatic |
| ACVR1B_c.1136+2T>C | S2 | 31.5 | 19.06 | 6.88 | 5013 | 4507 | 4000 | somatic |
| ACVR1B_c.85delG | S2 | 20.83 | 9.7 | 3.57 | 509 | 402 | 392 | somatic |
| ALK_c.1289C>A | S2 | 3.95 | 29.94 | 41.13 | 5421 | 4913 | 4872 | germline |
| ALK_c.4573A>G | S2 | 5.59 | 30.61 | 42.53 | 3705 | 3247 | 3348 | germline |
| APC_c.656C>T | S2 | 49.45 | 13.92 | 6.9 | 182 | 237 | 203 | somatic |
| ARAF_c.763delC | S2 | 86.78 | 42.2 | 13.99 | 3836 | 3019 | 3003 | somatic |
| ARID1A_c.2296dupC | S2 | 76.76 | 20.41 | 4.29 | 1437 | 2092 | 2567 | somatic |
| ARID1A_c.4892A>C | S2 | 7.83 | 2.89 | 0 | 3077 | 2837 | NA | somatic |
| ARID2_c.2806G>T | S2 | 38.83 | 43.23 | 44.73 | 6694 | 6591 | 5384 | germline |
| ARID2_c.5305C>T | S2 | 49.48 | 17.61 | 10.71 | 291 | 318 | 252 | somatic |
| AXL_c.379G>A | S2 | 9.85 | 4.64 | 0 | 5819 | 5777 | NA | somatic |
| B2M_c.43_44delCT | S2 | 83.34 | 30.75 | 9.51 | 7292 | 6049 | 6968 | somatic |
| BAX_c.121delG | S2 | 61.63 | 21.41 | 5.14 | 10684 | 10213 | 11553 | somatic |
| CARD11_c.2707G>A | S2 | 27.27 | 13.14 | 4.14 | 5468 | 3951 | 4030 | somatic |
| CD74_c.51G>A | S2 | 20.25 | 5.92 | 0 | 5738 | 4492 | NA | somatic |
| CDC73_c.968T>C | S2 | 45.45 | 13.73 | 5.69 | 814 | 772 | 808 | somatic |
| CDH1_c.2245C>T | S2 | 19.57 | 8.64 | 4.43 | 1242 | 1319 | 1219 | somatic |
| CDH1_c.2494G>A | S2 | 22.55 | 7.29 | 0 | 2333 | 2263 | NA | somatic |
| CREBBP_c.3250delA | S2 | 3.53 | 0 | 0 | 2494 | NA | NA | somatic |
| CREBBP_c.5488G>A | S2 | 41.77 | 15.87 | 4.49 | 12323 | 10252 | 12701 | somatic |
| CRKL_c.491G>A | S2 | 5.49 | 0 | 0 | 2077 | NA | NA | somatic |
| CSF1R_c.1497A>G | S2 | 8.76 | 2.89 | 0 | 2055 | 1659 | NA | somatic |
| DAXX_c.1884dupC | S2 | 7.85 | 3.91 | 0 | 1363 | 1354 | NA | somatic |
| ERBB2_c.838_839delinsTT | S2 | 41.99 | 17.59 | 5.03 | 4001 | 3717 | 3939 | somatic |
| ESR1_c.539A>G | S2 | 5.47 | 2.74 | 0 | 2415 | 3061 | NA | somatic |
| EXT1_c.369delA | S2 | 28.12 | 13.69 | 4.2 | 6953 | 5071 | 4481 | somatic |
| FAT1_c.3784C>T | S2 | 39.51 | 16.92 | 3.81 | 5270 | 4847 | 4934 | somatic |
| FAT1_c.8965delA | S2 | 8.5 | 5.57 | 2.61 | 1471 | 1347 | 1377 | somatic |
| GNAS_c.2153A>T | S2 | 26.93 | 10.86 | 3.33 | 1957 | 1556 | 1411 | somatic |
| JAK1_c.2580delA | S2 | 10.16 | 4.51 | 0 | 3023 | 2597 | NA | somatic |
| KLF4_c.709G>A | S2 | 18.59 | 10.08 | 3.53 | 8004 | 6481 | 7261 | somatic |
| MAP3K4_c.866A>G | S2 | 10.25 | 6.53 | 2.01 | 2058 | 2525 | 1994 | somatic |
| MSH2_c.2131C>T | S2 | 90 | 33.6 | 10.16 | 2449 | 3057 | 2421 | somatic |
| NF1_c.611T>C | S2 | 14.62 | 5.88 | 4.84 | 130 | 119 | 124 | somatic |
| NKX2-1_c.349A>G | S2 | 15.88 | 5.9 | 0 | 2292 | 1798 | NA | somatic |
| NOTCH1_c.1334C>T | S2 | 43.43 | 17.28 | 4.89 | 11362 | 9185 | 11422 | somatic |
| PALB2_c.1675_1676delinsTG | S2 | 6.02 | 0 | 0 | 980 | NA | NA | somatic |
| PDGFRB_c.2972G>A | S2 | 47.45 | 45.59 | 46.5 | 7812 | 7014 | 7721 | germline |
| PIK3CA_c.2308C>T | S2 | 38.79 | 24.41 | 4.9 | 348 | 295 | 286 | somatic |
| PIK3CA_c.3140A>G | S2 | 14.39 | 6.08 | 0 | 660 | 724 | NA | somatic |
| PRKCI_c.826delA | S2 | 11.6 | 2.78 | 0 | 957 | 899 | NA | somatic |
| RAF1_c.770C>T | S2 | 26.98 | 14.75 | 5.87 | 4337 | 4557 | 3953 | somatic |
| RET_c.1942G>A | S2 | 31.04 | 12.94 | 3.18 | 13619 | 11070 | 12644 | somatic |
| ROS1_c.1679G>A | S2 | 6.69 | 4.46 | 0 | 2273 | 2083 | NA | somatic |
| ROS1_c.4142-1G>A | S2 | 5.71 | 2.86 | 0 | 403 | 420 | NA | somatic |
| SALL4_c.1018G>A | S2 | 63.3 | 54.75 | 51.49 | 6714 | 4866 | 4700 | germline |
| SALL4_c.2996C>T | S2 | 28.49 | 13.47 | 4.36 | 5448 | 4144 | 3761 | somatic |
| SDHD_c.331G>A | S2 | 45.16 | 47.54 | 47.55 | 2263 | 2503 | 2105 | germline |
| SMO_c.1199G>A | S2 | 9.09 | 3.01 | 0 | 9964 | 7216 | NA | somatic |
| TERT_c.358C>T | S2 | 47.15 | 18.77 | 6.21 | 3334 | 2690 | 3093 | somatic |
| TP53_c.586C>T | S2 | 43.74 | 16.19 | 6.01 | 2835 | 2459 | 2747 | somatic |
| TSHR_c.457T>A | S2 | 15.21 | 2.97 | 0 | 743 | 809 | NA | somatic |
